# The Leydig Steroid Cell Tumor in a Postmenopausal Woman with Clinical and Biochemical Hyperandrogenism: A Case Report

**DOI:** 10.3390/metabo12070620

**Published:** 2022-07-04

**Authors:** Larisa V. Suturina, Eldar M. Sharifulin, Maharam A. Sharifulin, Ludmila M. Lazareva, Irina N. Danusevich, Kseniia D. Ievleva, Iana G. Nadeliaeva

**Affiliations:** 1 Federal State Public Institution “Scientific Center for Family Health and Human Reproduction Problems”, 16 Timiryazeva str., 664003 Irkutsk, Russia; sharifulja@mail.ru (E.M.S.); lirken_@mail.ru (L.M.L.); irinaemails@gmail.com (I.N.D.); asiy91@mail.ru (K.D.I.); ianadoc@mail.ru (I.G.N.); 2 Irkutsk Regional Clinical Hospital, 100 Yubileynyi, 664079 Irkutsk, Russia; masharifulin@mail.ru

**Keywords:** hyperandrogenism, postmenopause, Leydig cell tumors (LCTs), ovarian tumor, testosterone, hirsutism, case report

## Abstract

Leydig cell tumors (LCTs) refer to tumors of the stroma of the genital strand, which are found mainly in postmenopausal women. The diagnosis of LCTs in postmenopausal women is associated with specific difficulties and is based on the identification of hyperandrogenism with clinical manifestations of virilization, which has an erased picture in postmenopausal women. LCTs require differential diagnosis with other causes of hyperandrogenism. We present the clinical case of a 55-year-old Russian postmenopausal patient with LCTs of the right ovary, significantly increased levels of androgens, and rapidly progressive clinical signs of hyperandrogenism. The patient underwent laparoscopic bilateral salpingo-oophorectomy, and the androgen indices reached average values by the first and third month after surgery. This case demonstrates that LCTs are often benign with a good prognosis and normalization of the clinical and laboratory manifestations of hyperandrogenism after surgical treatment. The type of surgery performed (bilateral salpingo-oophorectomy rather than unilateral) is recommended as the treatment of choice for LCTs in postmenopausal patients.

## 1. Introduction

According to the WHO Classification, Leydig cell ovarian tumors (LCTs) are relatively rare sex cord stromal tumors [1], occurring mainly in postmenopausal ages.

Generally, hormonally active tumors account for about 5% of all ovarian masses [2,3,4], and the incidence of steroid cell tumors is less than 0.1% of all ovarian neoplasms [5]. Notably, there are associations between ovarian LCTs and gynecological cancer [6,7].

The diagnosis of LCTs in postmenopausal women is challenging [8]. Firstly, due to the rare incidence of LCTs, the OB-GYN and other specialists are typically not familiar with this group of ovarian tumors. Secondly, LCTs may be asymptomatic [9], and these patients often do not have enlarged ovaries when estimated by bimanual vaginal examination and pelvic ultrasound [10], especially in the absence of Doppler scanning. Diagnosis of LCTs in this group of patients is based on the detection of hyperandrogenemia and/or clinical manifestations of virilization, which often has an erased picture against the background of the natural aging processes in postmenopause [2,11,12,13]. LCTs require a differential diagnosis and the exclusion of other causes of hyperandrogenism [14].

The etiology and pathogenesis of LCTs as well as ovarian tumors in general are unknown. LCTs are usually benign with a good prognosis for normalization of clinical and laboratory manifestations of hyperandrogenism after surgical treatment [10,11,15,16].

The report aims to demonstrate a clinical case of a Leydig steroid cell tumor in a postmenopausal woman with late-onset, pronounced, rapidly progressive manifestations of clinical and biochemical hyperandrogenism.

## 2. Detailed Case Description

### 2.1. Clinical Information

This paper presents a clinical case of a 55-year-old Caucasian (Russian) postmenopausal patient with severe, progressive clinical signs of hyperandrogenism (hirsutism, acne, alopecia, oily seborrhea, baryphonia) over the past year before visiting a doctor. The first examination was carried out in July 2019 at the outpatient department of the Scientific Center for Family Health and Human Reproduction (Irkutsk, Russia). In our work, we followed the ethical principles established by the Declaration of Helsinki of the World Medical Association of 1964 (revised, Brazil, October 2013). Informed consent was obtained before the investigation. The patient also agreed to publish the anonymized data. This publication was approved by the Local Ethics Committee.

Complaints. Over the past year, the patient noted excessive hair growth along the upper lip, chin, lower abdomen, hair loss on the head, acne on the face and chest, alopecia, baryphonia, a sharp increase in body weight (15 kg over 3 months) without changing the diet, and the persistent rise in blood pressure in the absence of antihypertensive therapy.

Medical history. Operations: appendectomy (1973); cesarean section (1989); laparoscopic cholecystectomy (2009); hysteroresectoscopy; polypectomy (2017). Diseases: obesity since 2000 (ICD-10: E 66.0); type 2 diabetes since 2012 (ICD-10: E.11) (treatment: metformin 850 mg 2 times per day); atopic bronchial asthma since 2007 (ICD-10: J45.0) (treatment: salmeterol 25 mg/fluticasone propionate 250 mcg 2 doses 2 times per day, ipratropium bromide 0.25 mg/fenoterol bromyl 0.5 mg as needed); arterial hypertension since 2001 (ICD-10: I11.9) (treatment: telmisartan 80 mg 1 time per day, ivabradine 5 mg 2 times per day, torasemide 5 mg 1 time per day); hemorrhagic vasculitis (ICD-10: L95.8) since 1999 with an exacerbation in May 2019 (treatment: hydroxychloroquine sulfate 200 mg 2 tablets per day, pentoxifylline 100 mg 2 tablets 3 times per day). The patient had no bad habits or allergic reactions.

Gynecological History. The patient did not report any menstrual irregularities before menopause. Her menarche occurred at 13 years of age. She had 4 pregnancies (1: miscarriage of a short term; 2: caesarean section at 39 weeks, fetal weight 3400 g; 3−4: medical abortions). Contraception: intrauterine devices (3 times for 5 years each). The patient reached menopause at the age of 47 years without any severe climacteric manifestations. Gynecological diseases: uterine fibroids since 2013. The patient did not take antiandrogenic drugs or any medicines.

**Figure 1:**
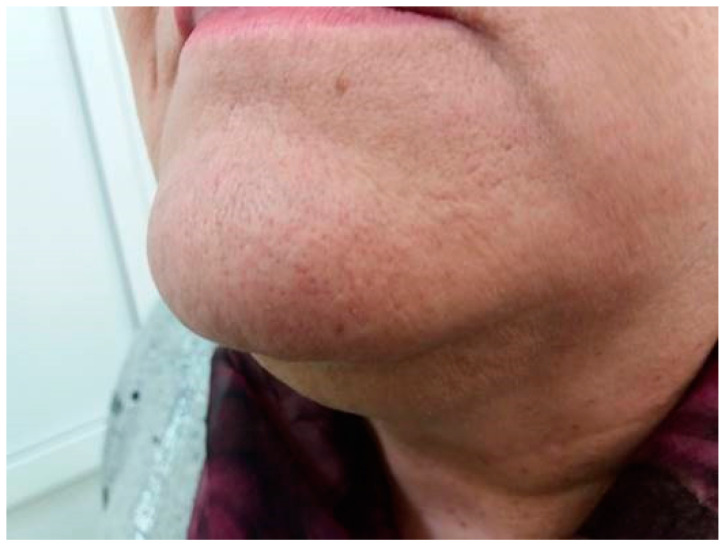
Patient before surgery. Ferriman−Gallwey score 5 (self-reported after shaving).

The anthropometric examination was carried out according to generally accepted methods. The patient had morbid obesity (BMI was 41.8 kg/m^2^). Blood pressure: 140/85 mmHg. Regarding hirsutism, the patient revealed excessive growth of terminal hair in androgen-dependent zones (upper lip, chin, lower abdomen, inner thighs), ranked 5 points by the Ferriman−Gallwey scale, according to her self-report because she applied shaving (Figure 1). Acne and seborrhea (face, chest, and back) as well as alopecia 1 degree, according to the Ludwig scale, were also revealed. Bimanual gynecological examination: no pathological changes were revealed.

Instrumental examination. Magnetic resonance imaging (MRI) was performed to exclude pituitary and extra pituitary masses. A multislice computed tomography (MCT) of the abdominal cavity, small pelvis, and retroperitoneal space with contrast (Aquilion One 640, Canon Medical Systems Corporation (formerly Toshiba Medical Systems), Ōtawara, Tochigi, Japan,) did not reveal pathological changes in the adrenal glands and ovaries as possible sources of hyperandrogenism.

Ultrasound examination of the adrenal glands, abdominal organs, and kidneys (ultrasound machine Aplio XG, Canon Medical Systems Corporation (formerly -Toshiba Medical Systems), Ōtawara, Tochigi, Japan), did not clarify the cause of hyperandrogenism. The results of the transabdominal ultrasound and transvaginal gynecological examination (Voluson E8, GE Healthcare, Chicago, IL, USA) were as follows: in the right ovary, 27 mm × 23 mm × 23 mm in size, focal masses that were 10.9 and 7 mm in size with pronounced blood flow were detected (for comparison: the left ovary measured 21 mm × 18 mm × 18 mm without follicles and active blood flow). Notably, the patient underwent transvaginal ultrasound examinations during the previous year, and no changes were detected.

Before surgery, examinations of the gastrointestinal tract (video esophagogastroduodenoscopy and video colonoscopy) were performed, and no pathological findings were detected. The endometrial Pipelle biopsy followed by a histological examination revealed fragments of the endometrial glands of the indifferent type.

Laboratory examination revealed high levels of total testosterone (TT), free (FT), and bioavailable testosterone (BT) whereas the levels of other hormones (including TSH, prolactin, cortisol, and insulin) were within the normative range (Table 1). The level of CA-125 was lower than the reference values (23.62 units/mL). When assessing the general clinical blood test, a pronounced blood concentration was found (RBC 5.42 × 10^12^/L, HCT 50.5%, HGB 162 g/L); other biochemical parameters were within normal limits, including fasting glucose (5.22 mM/L).

Based on the presented results of additional examination, the patient was diagnosed with hyperandrogenism (hirsutism, acne, alopecia, hyperandrogenemia) in postmenopause, most likely against the background of an androgen-producing tumor of the right ovary.

### 2.2. Intervention

**Figure 2:**
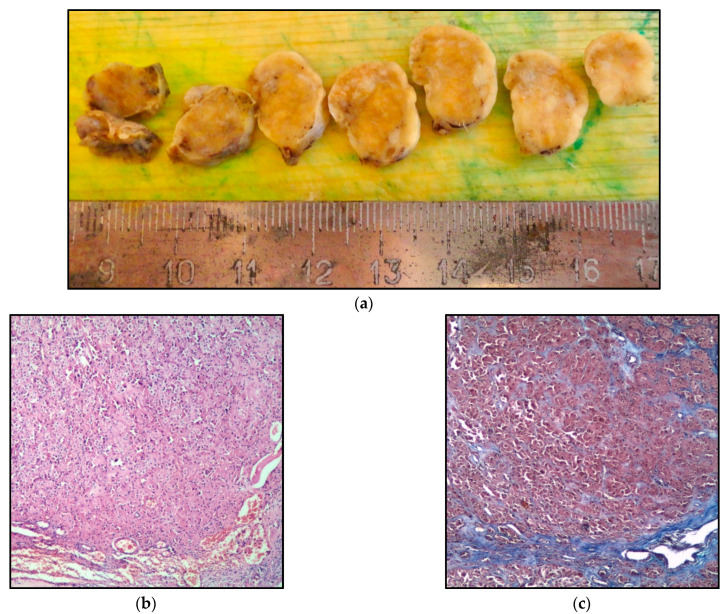

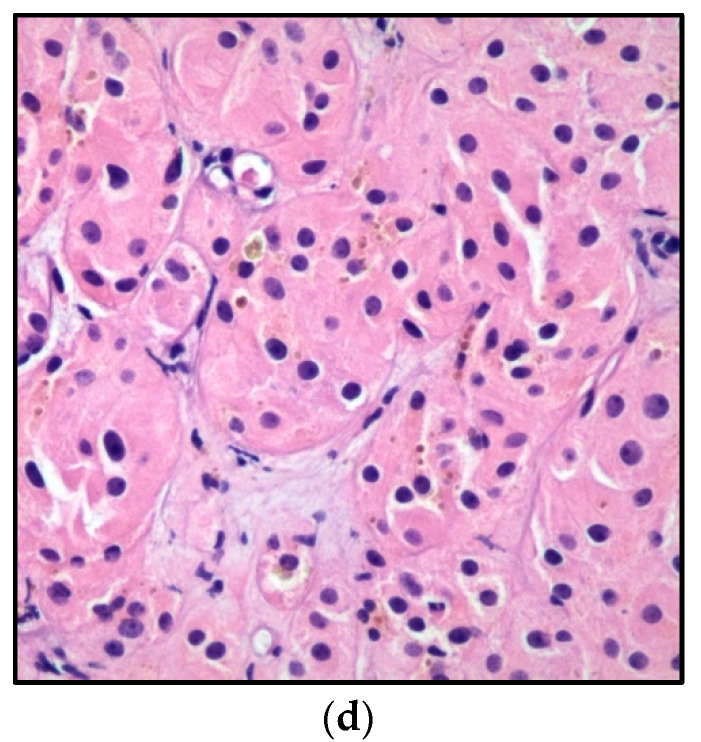
**Leydig cell tumors of the right ovary:** (**a**) **macro preparation**; (**b**) large fields of large round and polygonal cells, H&E, ×40; (**c**) weak fibrous stroma (collagen stromal fibers are colored blue), Masson’s trichrome, ×40; (**d**) tumor cells are round and polygonal with abundant fine-grained eosinophilic cytoplasm, H & E, ×200.

The patient underwent laparoscopic bilateral salpingo-oophorectomy, a biopsy of the omentum (at the gynecological department of the Irkutsk Regional Clinical Hospital, Irkutsk, Russia). The patient was discharged on the second day in a satisfactory condition. The result of the histological examination showed a steroid cell tumor of the right ovary from Leydig cells (Reinke’s crystals were absent) and focal hyperplasia of Leydig cells in the left ovary (Figure 2a–d).

### 2.3. After-Surgery Examination and Outcomes

To assess the clinical and laboratory manifestations of hyperandrogenism, the patient underwent a dynamic outpatient dispensary examination after 1 and 3 months with evaluation of complaints and physical and laboratory hormonal examinations, conducted at the Scientific Center for Family Health and Human Reproduction. Within a month, the patient’s acne and seborrhea improved, and by a 3-month follow-up, we registered a decrease in hirsutism to 2 points on the Ferriman−Gallwey scale and a decrease in body weight by 10 kg. In the hormonal test, 1 and 3 months after the surgery, androgen levels reached the reference values (all tests were performed in the same laboratory) (Table 1). We did not repeat the measurements of DHEAS, 17-OHP, prolactin, cortisol, and insulin at each visit because their levels at the baseline were within normal ranges.

## 3. Discussion

LCTs are sporadic tumors of the stromal sex cord of the ovary, most often occurring in postmenopausal women [17]. The etiology of the tumor is still unknown. However, we suppose that a certain contribution of previous morbid obesity to LCT development is not excluded. Recent experimental evidence suggests that tumor cells have estrogen receptors [18]. Simultaneously, obesity is often associated with hyperestrogenism. Thus, there is a theoretical basis for considering obesity in this study as an unfavorable factor. The above indicates the need for modification of lifestyle and nutrition, including the correction of gut microbiota, to prevent ovarian abnormalities influenced by obesity.

A key role in diagnosing this disease is played by a careful collection of clinical history and a detailed physical examination of these patients. LCTs can be suspected when a patient suddenly presents rapidly progressive clinical manifestations of hyperandrogenism. However, the clinical symptoms of the disease do not provide reliable information regarding the genesis of hyperandrogenism. Hormonal research has a significant diagnostic value [13]. A woman who demonstrates hyperandrogenism with increased serum testosterone levels beyond the normal range is often considered to have ovarian and adrenocortical tumors [19]. In this study, we observed increased TT, FAI, and BT whereas DHEAS levels were within regular intervals, corresponding to the previously reported cases of LCTs [20,21,22].

Hyperandrogenism in postmenopausal patients requires differential diagnosis, which includes hormone-producing tumors of the adrenal glands and ovaries, a nonclassical form of congenital adrenal cortical dysfunction, Cushing’s syndrome, and other causes (for example, medication intake). All these conditions were excluded in our patient.

Sex cord stromal tumors of the ovaries due to their small size (less than 4 cm) [2] and the absence of an increase in the size of the ovaries are often not visible without the use of highly sensitive instrumental diagnostic methods [23]. The ovarian mass was diagnosed in our case by a transvaginal pelvic ultrasound with the Doppler procedure. Transvaginal Doppler ultrasonography is very useful for detecting and diagnosing ovarian tumors [24]. However, conducting highly sensitive methods such as MSCT research and ultrasonography with dopplerography does not always have a positive diagnostic value in determining the etiology of hyperandrogenism in postmenopausal women. Instrumental methods have sensitivity limits, and the accuracy of the methods can also be affected by the qualifications of the doctor who conducts this study [10]. Several studies have shown the high resolution of MRI studies in tumors of the stroma of the ovarian sex cord. The sensitivity of this method was higher as compared to ultrasound in assessing postmenopausal hyperandrogenism [25]. The analysis of the presented clinical case compared with the literature data showed that only a comprehensive assessment and comparison of the disease’s clinical signs with hormonal and instrumental follow-up methods can achieve success in diagnosing LCTs.

After surgical treatment, the most cases of LCTs are characterized by a good correction of hyperandrogenism. It is proved that Leydig cells are not distributed locally but in more than 80% of the volume of ovarian tissue. It is generally accepted that LCTs should be determined if Leydig cell nodules exceed 1 cm in diameter while a size less than 1 cm is called Leydig cell hyperplasia; these two conditions are somewhat tricky to distinguish [26]. In the clinical case, we described a histological picture of Leydig cell hyperplasia in the second (left) ovary with an unpredictable prognosis of the disease. The type of surgery (bilateral oophorectomy) used in this study is explained by the previously described probability of hyperplasia and LCT in the contralateral ovary [27,28,29,30,31].

Study strength. Over the past decades, a relatively small number of clinical cases of LCTs and case series have been published [32,33,34,35,36]. Therefore, the demonstration of our observations is valuable and allows for replenishing the database available for pooled analysis. In our opinion, our study limitations are as follows: we did not measure IGF-1 when excluding acromegaly and did not perform a dexamethasone test or pelvic MRI.

## 4. Conclusions

The diagnosis of LCTs in postmenopausal women is associated with specific difficulties and requires differential diagnosis with other causes of hyperandrogenism. We have presented the clinical case of a 55-year-old Russian postmenopausal patient with LCTs of the right ovary and Leydig cell hyperplasia in the left ovary. After laparoscopic bilateral salpingo-oophorectomy, androgen indices reached average values by the first and third month. Therefore, this case supports that LCTs have a good prognosis after surgical treatment. The type of surgery performed (bilateral rather than unilateral salpingo-oophorectomy) is the treatment of choice for LCTs in postmenopausal patients because of the high likelihood of pathological changes in the contralateral ovary.

## Figures and Tables

**Table 1:** Serum concentrations of chosen hormones before and 1 and 3 months after surgical treatment.

	Before Surgery	After One Month	After Three Months	Reference Values
TT, nmol/L	7.6	0.95	0.67	0.22–2.9
FT, nmol/L	0.15	0.01	0.01	0.00–0.02
BT, nmol/L	3.59	0.47	0.26	0.09–0.29
SHBG, nmol/L	150.25	37.31	28.56	26.1–110.0
DHEAS, μmol/L	3.79	NA	NA	0.96–6.95
17-OHP, nmol/L	2.04	NA	NA	0.12–7.00
FSH, mIU/mL	64.86	53.23	63.59	25.80–134.8
Estradiol, pmol/L	196.9	90.34	70.58	<201.0
Prolactin, mIU/L	292.6	NA	NA	102.0–496
Cortisol, nmol/L	399.3	NA	NA	171.0–536.0
TSH, mIU/mL	1.5	NA	1.0	0.4–4.0
T4 free, pmol/L	17.8	NA	17.5	9.00–23.2
Fasting insulin, mcIU/mL *	18.7	NA	NA	2.6–24.9

Abbreviations: TT—total testosterone, FT—free testosterone, BT—bioavailable testosterone, SHBG—sex hormone binding globulin, 17-OHP—17-hydroxyprogesterone, DHEAS-dehydroepiandrosterone sulfate, FSH—follicle stimulating hormone, TSH—thyroid stimulating hormone, T4—thyroxine: NA (not available), while taking metformin *.

## Data Availability

Not applicable.

## Abbreviations

BMI	body mass index
BT	bioavailable testosterone
DHEAS	dehydroepiandrosterone sulfate
FSH	follicle-stimulating hormone
FT	free testosterone
LCTs	Leydig cell tumors
MRI	magnetic resonance imaging
MSCT	multislice computed tomography
T4	thyroxine
TT	total testosterone
TSH	thyroid-stimulating hormone
SHBG	sex hormone binding globulin
17-OHP	17-OH progesterone
